# Renal disease screening: a potential tool for reducing health inequity: Rastreamento para doença renal: uma ferramenta em potencial para reduzir a desigualdade na saúde

**DOI:** 10.1590/1516-3180.2016.13411512

**Published:** 2014-10-28

**Authors:** Paulo Andrade Lotufo

**Affiliations:** I MD, DrPH. Full Professor, Department of Internal Medicine, Faculdade de Medicina da Universidade de São Paulo (FMUSP), São Paulo, Brazil. Universidade de São Paulo Department of Internal Medicine Faculdade de Medicina Universidade de São Paulo São Paulo Brazil

The impact of chronic kidney disease on mortality, quality of life and cost of medical care is rising worldwide, such that it now affects 8-16% of the world population.^1^ The Global Burden of Diseases index, which ranks the causes of deaths, has revealed that as the underlying cause of death, chronic kidney disease (CKD) jumped from 34^th^ position in 1990 to 18^th^ in 2013.^2^ However, although the use of mortality statistics to ascertain temporal trends or make geographic comparisons has been helpful in charting other chronic diseases, this has not been the case for CKD. The reason for this is that when diabetes and CKD are mentioned together on a death certificate, the underlying cause is frequently stated as "diabetes without complications" and not "chronic kidney disease" or "diabetic kidney disease".^3^ Consequently, to ascertain the situation of CKD in countries like Brazil, other types of information are needed, such as registries, reports of medical procedures and epidemiological studies.^4^ ^5^ ^6^ ^7^

In Brazil, data from the National Dialysis Registry and the Ministry of Health have shown that over 10,000 people are currently undergoing kidney replacement therapy.^4^ de Moura et al. analyzed data from the Brazilian National Health System on 280,667 patients with end-stage renal disease who received publicly financed kidney replacement therapy for at least three consecutive months. Men (57.2%) and people aged 45-64 years (43.4%) were predominant. The underlying causes of CKD were hypertension (20.4%), diabetes (12.0%) and glomerulonephritis (7.7%). The annual increase in the prevalence of people under dialysis from 2000 to 2012 was 3.6% (95% confidence interval, CI: +3.2% to +4.0%) and the average annual change in incidence was +1.8%/year (+1.1% to +2.5%).^5^

The prevalence of CKD among apparently healthy people was analyzed among the participants of the Brazilian Longitudinal Study of Adult Health (ELSA-Brasil).^6^ ^7^ This was evaluated during baseline visits (2008-2010) to 14,636 civil servants aged 35 to 74 years. The definition of CKD based on albuminuria (albumin-to-creatinine ≥ 30 mg/g) and glomerular filtration rate < 60 ml/min/1.73 m^2)^. The glomerular filtration rate was obtained through an equation devised by the Chronic Kidney Disease Epidemiology Collaboration (CKD-EPI), which was derived from pooled data from clinical studies in the United States.^8^ In ELSA-Brasil, the frequency of albuminuria or low glomerular filtration rate, alone or in combination, was related to aging, lower socioeconomic status and self-reporting as black. Risk factors such as smoking, dyslipidemia, hypertension and diabetes were directly correlated with CKD. However, these risk factors did not explain the socioeconomic differences, i.e. the higher prevalence of CKD among the elderly, less affluent people and blacks.^9^

One point to emphasize is that ELSA-Brasil researchers did not correct the glomerular filtration rate for race in the manner proposed by the CKD-EPI consortium.^8^ The reason for their race correction was the greater mass muscle described in the African-American population. However, in contrast with research conducted in the United States, three independent studies in different Brazilian cities did not show that correction for black race was useful for the Brazilian population.^10^ ^11^ ^12^ These conclusions were the same as those relating to black people living in Ghana and South Africa.^13^ ^14^ In the light of these results in Brazil and Africa, there is no reason for medical laboratories in Brazil to keep on presenting glomerular filtration rates corrected for race.

Although the U.S. Preventive Services Task Force concluded that the "evidence is insufficient to assess the balance of benefits and harms of routine screening for CKD in asymptomatic adults,"^15^ we consider that creatinine measurements should be more widely used, so that glomerular filtration rates are not just estimated for people with hypertension or diabetes. As shown by the ELSA-Brasil results, socioeconomic status was inversely associated with CKD prevalence. Consequently, screening for CKD within primary care, especially at units located in places with poor and less educated populations, needs to evaluate this better: not only from a cost-effectiveness perspective but also from the perspective of the necessity to reduce inequity within the Brazilian population.
